# Using community science data to assess the association between urbanization and the presence of invasive *Aedes* species in Hungary

**DOI:** 10.1186/s13071-023-05780-7

**Published:** 2023-05-05

**Authors:** László Zsolt Garamszegi, Zoltán Soltész, Kornélia Kurucz, Tamara Szentiványi

**Affiliations:** 1 Institute of Ecology and Botany, Centre for Ecological Research, Alkotmány u. 2-4, Vácrátót, 2163 Hungary; 2 National Laboratory for Health Security, Centre for Ecological Research, Budapest, Hungary; 3 Institute of Biology, Faculty of Sciences, University of Pécs, Pécs, Hungary; 4 National Laboratory of Virology, Szentágothai Research Centre, University of Pécs, Pécs, Hungary; 5 Pathogen and Microbiome Institute, Northern Arizona University, Flagstaff, AZ USA

**Keywords:** Citizen science, Disease vector, Habitat preference, Urbanization level

## Abstract

**Background:**

Urbanization can be a significant contributor to the spread of invasive mosquito vector species, and the diseases they carry, as urbanized habitats provide access to a great density of food resources (humans and domestic animals) and offer abundant breeding sites for these vectors. Although anthropogenic landscapes are often associated with the presence of invasive mosquito species, we still have little understanding about the relationships between some of these and the built environment.

**Methods:**

This study explores the association between urbanization level and the occurrence of invasive *Aedes* species, specifically *Aedes albopictus*, *Aedes japonicus*, and *Aedes koreicus*, in Hungary, using data from a community (or citizen) science program undertaken between 2019 and 2022.

**Results:**

The association between each of these species and urbanized landscapes within an extensive geographic area was found to differ. Using the same standardized approach, *Ae. albopictus* showed a statistically significant and positive relationship with urbanization, whereas *Ae. japonicus* and *Ae. koreicus* did not.

**Conclusions:**

The findings highlight the importance of community science to mosquito research, as the data gathered using this approach can be used to make qualitative comparisons between species to explore their ecological requirements.

**Graphical Abstract:**

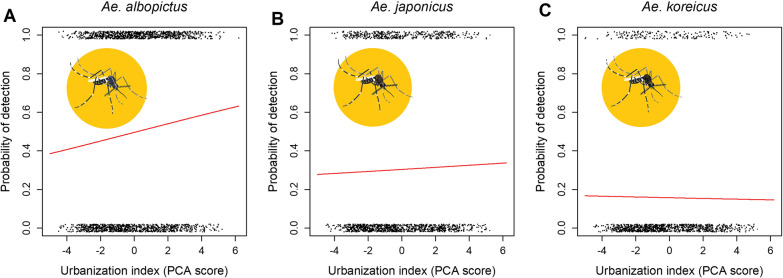

**Supplementary Information:**

The online version contains supplementary material available at 10.1186/s13071-023-05780-7.

## Background

Biological invasions are a global challenge as they can affect natural ecosystems in many ways and, in the case of newly introduced vector species, increase the potential for disease transmission. The current worldwide spread of invasive mosquito species, e.g. *Aedes aegypti*, *Aedes albopictus*, and *Anopheles stephensi*, is a major problem [1, 2], as some of them can transmit emerging and endemic pathogens of zoonotic and veterinary importance, including viruses, protozoans, and filarial nematodes [3].

Urbanization is a key environmental factor that can contribute to the emergence of certain invasive species and accelerate their spread across large geographical areas [4]. Urbanized habitats provide a wide variety of human-made breeding sites (e.g., stagnant water in cemeteries, artificial containers, catch basins, etc.). Invasive species can easily adapt to these habitats, where they may also find an unlimited source of food, including a high density of potential human and domestic animal hosts [5]. Furthermore, invasive species may also experience reduced competition in cities, which can affect their development and spread [6, 7]. The occurrence of certain invasive mosquito species, such as *Ae. aegypti* and *Ae. albopictus*, is often associated with heavily urbanized areas [4, 8–10], while comparable evidence for the habitat preference of other invasive species, such as *Aedes japonicus* and *Aedes koreicus,* is lacking or conflicting. A recent meta-analysis showed that the presence and/or abundance of *Ae. japonicus* was not affected by landscape anthropization (i.e., urbanization, agricultural activities, and deforestation), whereas a negative correlation was found for *Ae. koreicus* [10]. However, the meta-analysis included considerably fewer studies for these two species, thus inferences regarding the effect of urbanization on their distribution can be considered to have a weak basis. Furthermore, most of the available studies are based on the comparison of field-collected data between rural and urban sites or along an urban gradient [11, 12]. The number of sampling sites and the geographical scale of these studies are, however, usually limited due to the difficulties associated with performing wide-scale and standardized surveillance. Ideally, to gain a better understanding of the preference of different invasive species for urban habitats through comparison, one needs to monitor the presence of all of the species in parallel using the same standardized methodology and at the same geographic resolution.

Community (or citizen) science is a useful approach for the collection of occurrence data of invasive mosquito species on a wide spatial and temporal scale [13–15]. This approach is often more effective in detecting the occurrence of invasive mosquitoes than traditional surveillance methods [16]. Additionally, community science data appear to be repeatable, and prevalence and abundance estimates from community science programs seem to be as reliable as those collected during more conventional field studies [17].

The objective of this study was to determine the relationship between urbanization level and the presence of invasive mosquito species, specifically *Ae. albopictus*, *Ae. japonicus*, and *Ae. koreicus*, in Hungary, using occurrence data from community science.

## Methods

### Community science

Reports from the public on their encounters with suspected *Ae. albopictus, Ae. koreicus* and *Ae. japonicus* in an area of Hungary, Central Europe, covering ca. 93,030 km^2^, were submitted via email or mobile application (http://www.mosquitoalert.com) between 2019 and 2022 (http://www.mosquitosurveillance.hu/) [17]. Physical specimens sent by post were also considered for inclusion in the study. The observations were assessed by expert dipterologists, who identified the mosquitoes to species level using standard identification keys [17–19].

### Definition of variables

The validated reports were entered into a database, where each row reflected whether a citizen’s observation was a true report for any of the target invasive species (where 1 indicated that the taxonomic validation confirmed the detection of a target species and 0 indicated that it did not; these data were tabulated separately for each of the three species). Observations of mosquitoes of uncertain identity were removed from the data. Furthermore, for each target species, an entry was treated as missing if there was a confirmed observation of another target species in the same row.

Based on the geographic coordinates of the public observations, an urbanization index was calculated for the surrounding 1 × 1 km^2^ by using UrbanizationScore software [20]. This program scores the abundance of vegetation, buildings, and paved roads based on aerial images from Google Maps, and combines these information into a principal component that scores the locations along an urbanization gradient.

Each report included information on the date (year and day within a specific year, reflecting seasonal effects), and the mode (post, e-mail or mobile application) of each sample submission. These data were entered into the statistical model as control variables (see below).

### Statistical analyses

To explore the determinants of the probability of detection of the target species, a generalized linear model was fit with a binomial distribution and logit link function for each species separately. The response variable was the binary state variable, reflecting the detection of the given species in a given location, and the main predictor was the urbanization score. As control variables, the year and mode of sample submission were entered as categorical predictors, while the detection date was entered as a circular variable (using the sine and cosine of the radians of the actual date).

## Results

**Fig. 1:**
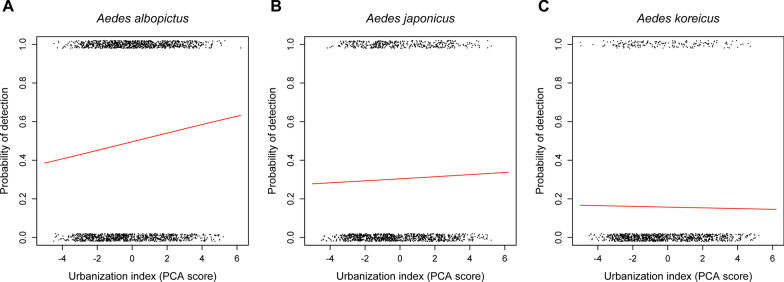
The effect of urbanization on the probability of detection for **a** *Aedes albopictus*, **b** *Aedes japonicus*, and **c** *Aedes koreicus*. Points indicate separate observations and are jittered along the* y*-axis for better visualization (sample sizes—*Ae. albopictus*,* n*_0_ = 983,* n*_1_ = 970; *Ae. japonicus*,* n*_0_ = 983,* n*_1_ = 428; *Ae. japonicus*,* n*_0_ = 983,* n*_1_ = 184). Lines are regression lines calculated from the appropriate generalized linear model.* PCA* Principal component analysis

The number of confirmed observations for each of the invasive species was systematically lower than the number of reports for the native species (see species-specific sample sizes in Fig. 1). These sample sizes indicated that a single observation of an invasive species an be matched with one to five observations of noninvasive species as controls, thus the sample was considered to be sufficiently balanced. Urbanization scores were normally distributed (mean ± SE, − 0.004 ± 0.040; range, − 5.013–6.202), indicating that the citizen observations were for a wide range of habitat types, and that the data were not biased with respect to reports from cities (Table 1).

**Table 1:** The relationship between the probability of detection of *Aedes albopictus* and the level of urbanization, while controlling for year and seasonal effects and the mode of sample submission in a generalized linear model (with binomial distribution)

Response: true vs false observations for *Aedes albopictus*		
	β (± SE)	*P*
Intercept	− 3.483 (± 0.372)	> 0.001
Urbanization score	0.157 (± 0.030)	> 0.001
Sine(radian date)	− 0.336 (± 0.144)	0.020
Cosine(radian date)	− 3.963 (± 0.243)	> 0.001
Year (2020)	0.197 (± 0.197)	0.316
Year (2021)	2.130 (± 0.226)	> 0.001
Year (2022)	3.498 (± 0.276)	> 0.001
Mode of submission (package)	0.138 (± 0.519)	0.790
Mode of submission (email)	− 0.316 (± 0.249)	0.206

Data are from a community science program undertaken in Hungary. Results for *Aedes japonicus* and *Aedes koreicus* are shown in Additional file 1: Table S1

*P*-values correspond to α = 0.05

The generalized linear models revealed that, when controlling for the potentially confounding variables, the urbanization index was positively associated with the occurrence of *Ae. albopictus* (Fig. 1a; Additional file 1: Table S1). However, the urbanization score was not a significant predictor of the presence of *Ae. japonicus* or *Ae. koreicus* (Fig. 1b, c; Additional file 1: Table S1). The estimates for the control variables indicated that year and seasonal effects were significant predictors for the detection of the target species and, in the case of *Ae. japonicus*, that the mode of sample submission was also a significant predictor (Additional file 1: Table S1).

## Discussion

The findings on *Ae. albopictus* support the predictions and results of previous studies regarding the preference of this species for urban habitats [5, 10, 21]. The difference between the current study and previous ones, however, is that it relied, to our knowledge for the first time, on community science to provide this evidence. The results of the present study also confirm previous findings on *Ae. japonicus* that showed no association of this species with urbanization, which suggests that this species exploits urban and rural areas equally [11, 22]. Neither did the occurrence of *Ae. koreicus* show a clear association with the level of urbanization, which contrasts with theoretical expectations [23–25] and the findings of a recent meta-analysis that showed a negative association between this species and urban environments [10].

As the current study applied the same standard methodology for the three focal species, the obtained effect sizes are comparable. Thus, the results obtained allowed for quantitative comparisons between the three invasive species. Although invasive species are generally assumed to be associated with urbanized habitats [26], the results of this work indicate that the association between species occurrence and level of urbanization can vary between closely related species, even within the same geographical area. Considering that the focal species were first detected in Hungary about a decade ago and have had approximately the same amount of time to disperse [24, 27], the results also indicate that different mosquito species may respond differently to the same ecological constraints during this process.

It has been suggested that *Ae. albopictus* has a preference for humans when it is present in non-native areas—and may also occasionally feed on other available mammals or even birds, including domestic animals—which may partially explain its strong association with urbanized habitats [28]. However, a preference for urban habitats may also evolve as a result of enriched conditions for breeding in these artificial environments, and human biting behavior may actually be a secondary consequence of this environment-mediated adaptation [29]. Urban and peri-urban sites can offer favorable microclimatic factors for breeding [30], an increased number of breeding sites [31], improved water quality [32], and even a reduced chance of the co-occurrence of competitive species and predators [33–35]. Given the correlative nature of the available evidence, it remains unknown whether the association of *Ae. albopictus* with urbanized landscapes is mediated by breeding or foraging ecology or, to some extent, both. Note that *Ae. japonicus* and *Ae. koreicus* exhibit largely mammalophilic feeding behavior, with occasional biting on humans [28, 36], and that they are typically found in natural or rural environments [11, 37, 38].

It is important to note that, although community science appears to be a useful tool for the surveillance of invasive mosquitoes, it has its own limitations. Findings based on this approach can be confounded by, for example, non-random sampling, underrepresented geographical areas, unbalanced sampling effort, and requirements for data validation [39, 40]. However, these shortcomings were unlikely to have caused a considerable degree of bias in the current study, as the distribution of the data suggested that localities were randomly sampled along an urbanization gradient between two extremes, irrespective of the underlying sampling effort (Fig. 1). Depending on the research question, future studies should also consider including traditional collection methods, and ideally apply direct field sampling in parallel with indirect surveillance based on community science.

## Conclusions

These results provide novel insights into the distribution and habitat preferences of invasive mosquito species, and highlight the importance of community science in the field of mosquito research and control. The results indicate that community science may not only be useful for tracking the spread and occurrence of invasive species, but may also allow qualitative comparison between species to further explore their ecological requirements. The use of a unified and standard methodology allowed the identification of species-specific roles that affected the spread of three invasive mosquito species in the same country within the same time frame.

## Supplementary Information

**Additional file 1: Table S1.** Results of the generalized linear model.

**Additional file 2: Dataset S1. **Urbanization scores and the probability of detection (0 or 1) for *Aedes albopictus*, *Aedes japonicus* and *Aedes koreicus* in Hungary, as determined using the data of a community science survey undertaken between 2019 and 2022.

## Data Availability

All the data generated or analyzed during this study are included in this published article (and Additional file 2: Dataset S1).
