# Correction: Using community science data to assess the association between urbanization and the presence of invasive Aedes species in Hungary

**DOI:** 10.1186/s13071-023-05818-w

**Published:** 2023-06-01

**Authors:** László Zsolt Garamszegi, Zoltán Soltész, Kornélia Kurucz, Tamara Szentiványi

**Affiliations:** 1 Institute of Ecology and Botany, Centre for Ecological Research, Alkotmány U. 2-4, Vácrátót, 2163 Hungary; 2 National Laboratory for Health Security, Centre for Ecological Research, Budapest, Hungary; 3 Institute of Biology, Faculty of Sciences, University of Pécs, Pécs, Hungary; 4 National Laboratory of Virology, Szentágothai Research Centre, University of Pécs, Pécs, Hungary; 5 Pathogen and Microbiome Institute, Northern Arizona University, Flagstaff, AZ USA

**Correction: Parasites & Vectors (2023) 16:158** 10.1186/s13071-023-05780-7

Following publication of the original article [1], it came to the authors' attention that the article had published with an error in Additional file 2: the column 'urbanization score' was missing from the file. The file has since been corrected in the published article. The authors thank you for reading this erratum and apologize for any inconvenience caused.
