# Supplementary material for: Using community science data to assess the association between urbanization and the presence of invasive Aedes species in Hungary
Source: Parasit Vectors. 2023 May 5;16:158. doi: 10.1186/s13071-023-05780-7 (PMC10161419; doi:10.1186/s13071-023-05780-7)
Supplement: Supplementary file 1 — Additional file 1: Table S1. Results of the generalized linear model. [file 13071_2023_5780_MOESM1_ESM.docx]

*Additional Material to*

**Using community science data to assess the association between urbanization and the presence of invasive *Aedes* species in Hungary**

László Zsolt Garamszegi^1,2*^, Zoltán Soltész^1^, Kornélia Kurucz^3,4^, Tamara Szentivanyi^1,5^

^1^Institute of Ecology and Botany, Centre for Ecological Research, Vácrátót, Hungary

^2^National Laboratory for Health Security, Centre for Ecological Research, Budapest, Hungary

^3^Institute of Biology, Faculty of Sciences, University of Pécs, Pécs, Hungary

^4^National Laboratory of Virology, Szentágothai Research Centre, University of Pécs, Pécs, Hungary

^5^Pathogen and Microbiome Institute, Northern Arizona University, Flagstaff, AZ, USA

**Table S1**. The relationship between the probability of detection of two invasive mosquito species (*Ae. japonicus* and *Ae. koreicus*) and the level of urbanization while controlling for year and seasonal effects and the mode of sample submission in a generalized linear model (with binomial distribution). Data are from a community science program run in Hungary; P values correspond to α = 0.05.

| **Response:** True vs. false observations for the species | | |
| --- | --- | --- |
| ***Ae. japonicus*** | *β* (± SE) | *P* |
| Intercept | -3.990 (± 0.474) | <0.001 |
| Urbanisation score | 0.045 (± 0.028) | 0.118 |
| *sin*(radian date) | -0.006 (± 0.127) | 0.965 |
| *cos*(radian date) | -1.491 (± 0.181) | <0.001 |
| Year [2020] | 0.593 (± 0.178) | 0.001 |
| Year [2021] | 1.273 (± 0.218) | <0.001 |
| Year [2022] | 1.869 (± 0.250) | <0.001 |
| Mode of submission [package] | 1.070 (± 0.674) | 0.113 |
| Mode of submission [email] | 1.692 (± 0.415) | <0.001 |
| ***Ae. koreicus*** | β (± SE) | *P* |
| Intercept | -4.745 (± 0.590) | <0.001 |
| Urbanisation score | -0.002 (± 0.042) | 0.969 |
| *sin*(radian date) | -0.177 (± 0.189) | 0.349 |
| *cos*(radian date) | -1.648 (± 0.218) | <0.001 |
| Year [2020] | 1.300 (± 0.479) | 0.007 |
| Year [2021] | 2.709 (± 0.491) | <0.001 |
| Year [2022] | 4.172 (± 0.494) | <0.001 |
| Mode of submission [package] | 1.154 (± 0.615) | 0.061 |
| Mode of submission [email] | 0.166 (± 0.344) | 0.629 |
